# BRICS sequential therapeutic regimen as first-Line treatment for PD-L1-negative metastatic non-small cell lung cancer patients harboring EGFR/ALK wild-type status: a retrospective study

**DOI:** 10.3389/fimmu.2025.1618110

**Published:** 2025-06-23

**Authors:** Jianxin Chen, Jian Wang, Weiqiang Fang, Yating Wu, Hang Li, Hui Xu, Yunyun Zhu, Yanran Cheng, Zongyang Yu, Yonghai Peng

**Affiliations:** ^1^ M.D. Department of Education, International Word, The Quzhou Affiliated Hospital of Wenzhou Medical University, Quzhou People’s Hospital, Quzhou, Zhejiang, China; ^2^ M.D. Department of Gastroenterology, Jiaxing Second Hospital, Jiaxing, Zhejiang, China; ^3^ Department of Oncology, Cangshan Hospital Area, 900 Hospital of the Joint Logistics Support Force, Fujian, China

**Keywords:** BRICS regimen, NSCLC, first-line treatment, retrospective study, efficacy

## Abstract

**Background:**

Patients with PD-L1-negative, EGFR/ALK wild-type metastatic non-small cell lung cancer (NSCLC) exhibit limited responses to immune checkpoint inhibitors (ICIs). This study evaluates the BRICS regimen-a sequential approach combining stereotactic body radiotherapy (SBRT), probiotics, PD-1 inhibitors, and low-dose chemotherapy-to overcome immunotherapy resistance.

**Methods:**

This retrospective study included 23 patients treated between 2018 to 2024. Eligibility criteria: confirmed PD-L1-negative NSCLC, no actionable mutations, and measurable lesions. The BRICS regimen comprised SBRT (24 Gy in 3 fractions) to a single lesion, oral probiotics (6 g/day), low-dose chemotherapy, and PD-1 inhibitors administered every 21 days for six cycles. Outcomes included objective response rate (ORR), disease control rate (DCR), progression-free survival (PFS), overall survival (OS), and safety.

**Results:**

Median age was 62 years; 82.6% were male. ORR and DCR were both 95.7%. Median PFS was 16 months (95% CI: 9.11-22.89), and median OS was 32.7 months (95% CI: 11.53-53.87). In subgroup analysis based on prior treatment status, median PFS and OS were numerically longer in treatment-naïve patients compared to previously treated patients (mPFS: 20.0 vs. 13.6 months; mOS: 48.0 vs. 18.0 months), though without statistical significance (P > 0.05). Poor ECOG performance status predicted poorer PFS (HR=9.908, p=0.013) and OS (HR=26.406, p=0.008). Adverse events were predominantly grade 1 to 2 (fatigue:13.2%, rash:8.7%), with no grade ≥3 toxicities.

**Conclusions:**

The BRICS regimen demonstrated promising efficacy and safety in PD-L1-negative NSCLC, potentially overcoming resistance through multimodal immunomodulation. clinical benefit was observed regardless of treatment line, with a trend toward improved outcomes when administered as first-line therapy. Prospective trials are warranted to validate these findings and explore mechanisms underlying radiotherapy–microbiome–chemotherapy synergy.

## 1 Introduction

Lung cancer is the leading cause of cancer-related deaths and represents one of the most significant public health challenges worldwide (1). The American Cancer Society (ACS) estimates that by 2025, there will be 226,650 new cases and 124,730 deaths attributed to lung cancer in the United States (2). Non-small cell lung cancer (NSCLC) is the most prevalent type of lung-associated malignancy, accounting for approximately 85% of all lung cancer cases, with about 70% diagnosed as non-squamous pathological types such as adenocarcinoma or large cell carcinoma (3). More than half of newly diagnosed patients are considered incurable due to the presence of metastases at the time of initial presentation (4).

For patients with advanced or metastatic NSCLC, the National Comprehensive Cancer Network (NCCN) guidelines recommend molecular testing to identify treatable target mutations, including KRAS, EGFR, MET, ALK, and ROS1 (5). Most patients harboring these mutations can achieve varying degrees of response through targeted therapies. In cases of advanced NSCLC without driver genes, first-line treatment is determined by PD-L1 expression levels and histological type. For patients exhibiting high PD-L1 expression levels (≥50%), single-agent pembrolizumab is recommended. Conversely, for patients with low PD-L1 expression (tumor proportion score of 0%–49%), the standard treatment involves platinum-based chemotherapy in combination with immunotherapy (6). According to a global multicenter study aimed at verifying the real-world prevalence of PD-L1 expression in locally advanced or metastatic NSCLC, nearly half of the patients were found to be PD-L1 negative (7). A real-world study investigating the actual effects of combined chemotherapy and PD-1/PD-L1 inhibitors in the treatment of advanced NSCLC revealed that patients with negative PD-L1 expression experienced poorer prognoses following combined treatment (8). Notably, this population is characterized by an extremely low response rate to immunotherapy. Therefore, despite the recent updates in guidelines indicating that the addition of immunotherapy has improved the prognosis for patients with advanced NSCLC, those with negative PD-L1 expression continue to face limited OS, significant toxic side effects, and challenges related to immune resistance. Consequently, exploring new combination therapy strategies to overcome the treatment bottleneck for PD-L1-negative patients and reduce adverse reactions has become a focal point of research within the global oncology community.

Recent advances in cancer immunotherapy have illuminated several promising strategies to overcome PD-(L)1 inhibitors resistance in immunologically “cold” tumors. Radiation therapy, particularly hypo-fractionated regimens, has demonstrated immunomodulatory effects through induction of immunogenic cell death, release of tumor neoantigens, and enhancement of dendritic cell cross-presentation. The phase II PEMBRO-RT trial revealed that stereotactic body radiotherapy (SBRT) combined with pembrolizumab doubled objective response rates in PD-L1-negative NSCLC compared to immunotherapy alone (9). Concurrently, emerging evidence underscores the critical role of gut microbiota in modulating immune checkpoint inhibitor (ICI) responses. Seminal studies by Gopalakrishnan et al. (10) and Matson et al. (11) identified specific Bifidobacterium species as potent enhancers of anti-PD-1 efficacy through CD8+ T cell activation and dendritic cell maturation. Clinical translation of these findings was demonstrated in the other researches, where increased the efficacy of immunotherapy in solid cancers (12–15).

The potential synergy between radiotherapy, microbiome modulation, and metronomic chemotherapy has garnered increasing attention. Low-dose chemotherapy regimens have shown unique immunostimulatory properties, including depletion of regulatory T cells (Tregs) and myeloid-derived suppressor cells (MDSCs), while preserving effector T cell populations (16, 17). A phase II study reported that combining low-dose paclitaxel with ICIs improved progression-free survival (PFS) and OS in platinum-refractory urothelial carcinoma (18). Building upon these scientific premises, our center has developed the BRICS sequential therapeutic regimen - an acronym derived from Bifidobacterium supplementation, Radiotherapy (hypofractionated), Immunotherapy (PD-1 inhibitors), Chemotherapy (low-dose), and Stereotactic approach. This multimodal strategy aims to sequentially: 1) prime the TME through radiation-induced antigen release, 2) enhance systemic immunity via probiotic-mediated gut-immune axis modulation, 3) sustain tumor control with low-dose chemotherapy’s immunogenic effects, and 4) amplify immune recognition through PD-1/PD-L1 blockade.

The present retrospective study was thereby conducted to investigated the efficacy and safety of the BRICS sequential therapeutic regimen in PD-L1-negative metastatic non-small cell lung cancer patients harboring EGFR/ALK wild-type status.

## 2 Methods

### 2.1 Data source

The data of patients diagnosed as metastatic NSCLC with EGFR/ALK wild-type, PD-L1-negative at Cangshan Hospital Area, 900 Hospital of the Joint Logistics Support Force, and Quzhou People’s Hospital between January 2018 and December 2024 were retrieved from the electronic medical record system. Patients were deemed eligible for inclusion in this retrospective real-world study if they met the following criteria: (1) a definitive histological or cytological diagnosis of advanced NSCLC; (2) confirmed negative PD-L1 expression; (3) absence of treatable target mutations; (4) receipt of the ‘BRICS’ treatment strategy; and (5) presence of at least one measurable lesion. In addition, the exclusion criteria were as follows: (1) a history of autoimmune disease; (2) a poor ECOG performance status of > 2. (3). without measurable lesions. The follow-up deadline was set for December 31, 2024. This study received approval from the Ethics Committee of Quzhou People’s Hospital and the Ethics Committee of 900 Hospital of the Joint Logistics Support Force, and all investigations were conducted in accordance with the Declaration of Helsinki (revised in 2013).

### 2.2 Treatment procedure

**Figure 1:**
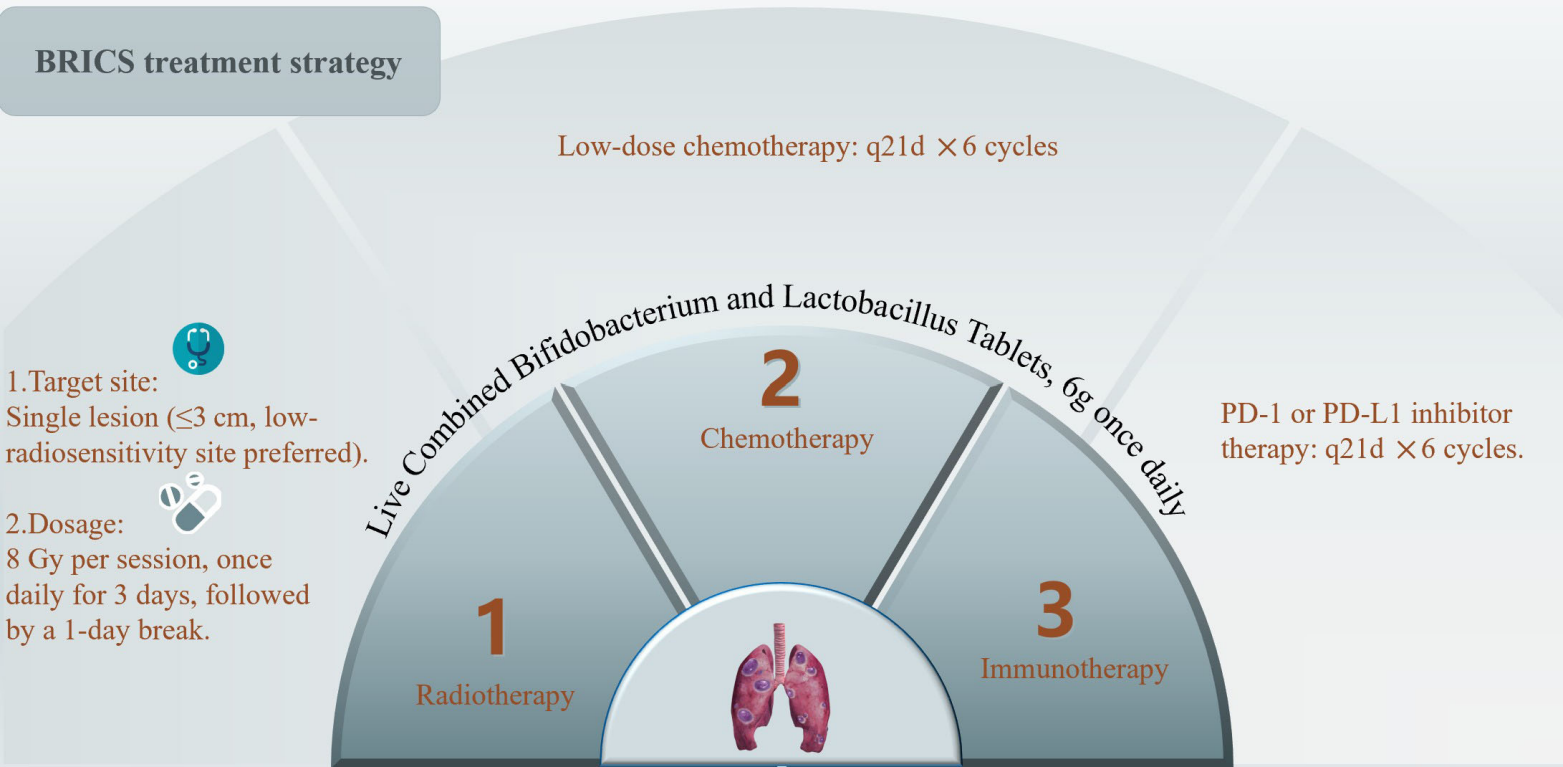
Treatment protocol of BRICS sequential therapeutic regimen.

The BRICS therapeutic regimen was follows: Eligible patients underwent stereotactic body radiotherapy (SBRT) targeting a single metastatic lesion (preferably ≤3 cm, anatomically well-circumscribed, and located in low-radiosensitivity regions such as peripheral lung parenchyma) with 8 Gy delivered daily over three consecutive days (total 24 Gy). Concurrently, oral triple-dose Bifidobacterium/Lactobacillus probiotics (6 g/day) were initiated and continued indefinitely. After a 24-hour post-SBRT recovery period, patients received low-dose chemotherapy (such as nab-paclitaxel 200 mg intravenously) combined with a PD-1 inhibitor on day 5, repeated every 21 days for six cycles. Chemotherapy dose reductions (to 150 mg) were implemented for grade ≥3 hematologic toxicity, while immune-related adverse events were managed per ASCO guidelines. Following completion of six cycles, patients continued probiotic maintenance while undergoing quarterly surveillance with contrast-enhanced CT/MRI monitoring. In cases of disease progression, a new cycle of BRICS therapy was initiated by selecting one progressing lesion meeting the original SBRT criteria (≤3 cm, non-critical location) for re-irradiation (8 Gy ×3 fractions). Post-SBRT, the patient received an additional six cycles of BRICS chemoimmunotherapy (nab-paclitaxel + PD-1 inhibitor), followed by treatment discontinuation and surveillance. This iterative approach continued until systemic progression or intolerance, with cumulative radiation doses constrained to organ-at-risk tolerance limits (e.g., spinal cord cumulative Dmax <45 Gy). The protocol prioritized temporal coordination of SBRT-induced antigen release, microbiome modulation, and metronomic chemotherapy to sustain immune activation. The treatment protocol was presented in the **Figure 1**.

### 2.3 Data collection and outcomes evaluations

Clinical response to the BRICS sequential therapeutic regimen was evaluated according to the Response Evaluation Criteria in Solid Tumors (RECIST) version 1.1. The enrolled patients underwent imaging evaluation every 6–8 weeks during treatment, while every 12 weeks after treatment. The objective response rate (ORR) was defined as the percentage of patients who achieved a complete response (CR: complete remission of all target lesions) or partial response (PR: at least a 30% reduction in the sum of the diameters of target lesions). Progressive disease (PD) referred to a 20% increase in the sum of the diameters of target lesions. A disease that could not be classified as PR or PD was evaluated as stable disease (SD). The percentage of patients with CR, PR, or SD was defined as the disease control rate (DCR). Duration of response (DoR) refers to the time from the first PR to the first PD among patients who had been evaluated as PR. PFS was calculated as the time from the initiation treatment of BRICS to PD or death. OS referred to the time from the initiation of anti-PD-1 inhibitor treatment to PD or death. Adverse events (AEs) were graded according to the National Cancer Institute Common Terminology Criteria for Adverse Events version 4.0 (NCI-CTCAE v4.0).

### 2.4 Statistical analysis

Descriptive statistics (percentages, means, and medians) were used to describe the baseline characteristics and clinical features of the patients with advanced NSCLC. Short-term efficacy was evaluated using ORR and DCR. Survival curves were calculated using the Kaplan-Meier method and were compared via the log-rank test based on ECOG PS. K-M curves were plotted using GraphPad Prism 9.0 (GraphPad Software Inc., San Diego, CA, USA). These analyses were performed using SPSS software, version 23.0 (SPSS Inc., Chicago, USA). P ≤ 0.05 was considered to indicate statistical significance.

## 3 Results

### 3.1 Patient characteristics and outcomes

A total of 23 patients were included in this study. The characteristics of the patients are summarized in **Table 1**. The median age was 62.0 years, and the histological type was adenocarcinoma. Most patients were male (82.6%), had a history of smoking (69.6%), and were classified as TNM stage IV (78.3%). The number of metastatic organs was as follows: 0 (21.7%), 1 (34.8%), and ≥2 (43.5%). Notably, 47.8% of patients did not receive first-line treatment, while 43.5% received first-line treatment, and 8.7% received second-line treatment. Additionally, 91.3% of patients had a PS score of <2. Only 2 patients (8.7%) received immunotherapy before BRICS treatment. Among the patients, 69.6% selected the lung as the site for radiotherapy, and 87% chose nab-paclitaxel as their chemotherapy agent. The immunotherapy drugs selected were predominantly Toripalimab (47.8%) and Camrelizumab (39.3%).

**Table 1:** Baseline characteristics.

Baseline characteristics	All patients (n = 23)
Age (years), n (%)	
Median (range)	62 (54-67)
≥60	15 (65.2)
<60	8 (34.8)
Gender, n (%)	
Male	19 (82.6)
Female	4 (17.4)
TNM stage, n (%)	
III	5 (21.7)
IV	18 (78.3)
Smoking status, n (%)	
Non-smoker	16 (69.6)
Former smoker/smoker	7 (30.4)
Number of metastatic organs, n (%)	
0	5 (21.7)
1–2	8 (34.8)
≥ 2	10 (43.5)
ECOG PS, n (%)	
0–1	21 (91.3)
2	2 (8.7)
Number of prior lines of standard therapy, n (%)	
First-line therapy	10 (43.5)
Second-line therapy	2 (8.7)
No prior therapy	11 (47.8)
History of PD-1/PD-L1 inhibitor treatment in prior therapy, n (%)	2 (8.7)
Irradiated organs in the BRICS regimen, n (%)	
Brain	1 (4.3)
Lung	16 (69.6)
Bone	1 (4.3)
Liver	3 (13.2)
Adrenal glands	1 (4.3)
Pleura	1 (4.3)
Chemotherapy agents in the BRICS regimen, n (%)	
Nab-paclitaxel	20 (87.0)
Pemetrexed	2 (8.7)
Lobaplatin	1 (4.3)
Immunotherapy agents in the BRICS regimen, n (%)	
Camrelizumab	9 (39.3)
Sintilimab	1 (4.3)
Toripalimab	11 (47.8)
Tislelizumab	1 (4.3)
Pembrolizumab	1 (4.3)
Number of BRICS regimen treatment cycles, n (%)	
6	7 (30.4)
4-6	16 (69.6)

ECOG PS, eastern cooperative oncology group performance status; PD-1, programmed cell death protein 1; BRICS, Bifidobacterium supplementation, Radiotherapy, Immunotherapy (PD-1 inhibitors), Chemotherapy, and Stereotactic approach.

### 3.2 Clinical outcomes

**Figure 2:**
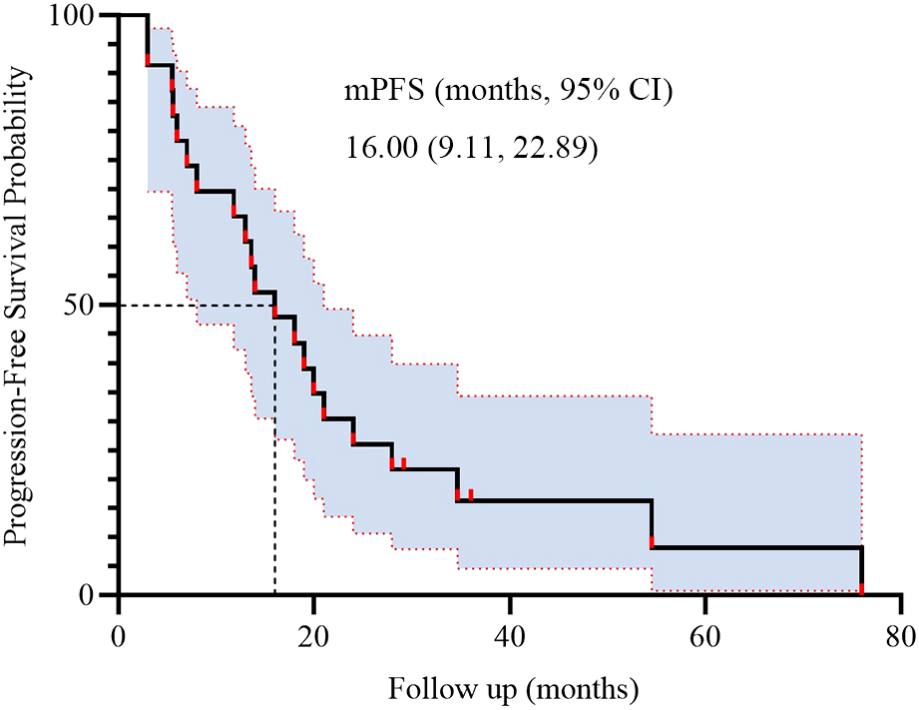
Kaplan-Meier survival curves of PFS in 23 patients.

**Figure 3:**
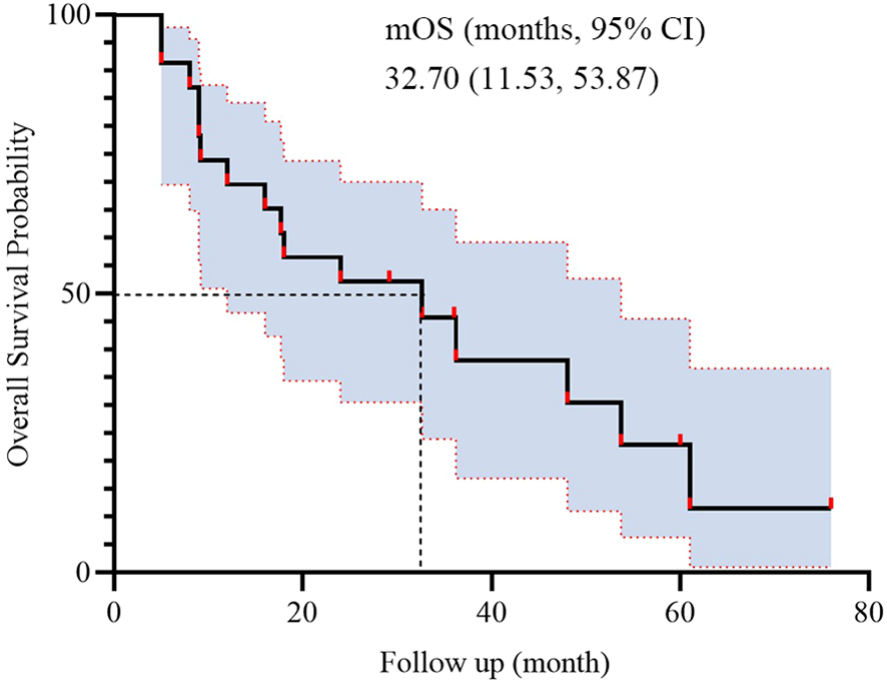
Kaplan-Meier survival curves of OS in 23 patients.

During BRICS treatment, all patients underwent regular imaging reviews. As shown in **Table 2**, partial response (PR) was observed in 17 of the 23 patients (74.0%), stable disease (SD) in 5 patients (21.7%), and progressive disease (PD) in 1 patient. Both disease control rate (DCR) and overall response rate (ORR) were 95.7%. The median progression-free survival (PFS) was 16 months, with a 95% confidence interval (CI) of 9.11 to 22.89 months (**Figure 2**). The median overall survival (OS) was 32.7 months, with a 95% CI of 11.53 to 53.87 months (**Figure 3**).

**Table 2:** Efficacy of BRICS regimen in advanced NSCLC patients (n = 23).

Efficacy	All patients (n = 23)
Complete response (%)	0
Partial response (%)	17 (74.0)
Stable disease (%)	5 (21.7)
Progressive disease (%)	1 (4.3)
Objective response rate (%, CR, PR)	17 (74.0)
Disease control rate (%, CR, PR, SD)	22 (95.7)
median progression-free survival (months, 95% CI)	16.00 (9.11, 22.89)
median Overall Survival (months, 95% CI)	32.70 (11.53, 53.87)

### 3.3 Prior therapy subgroup analysis

**Figure 4:**
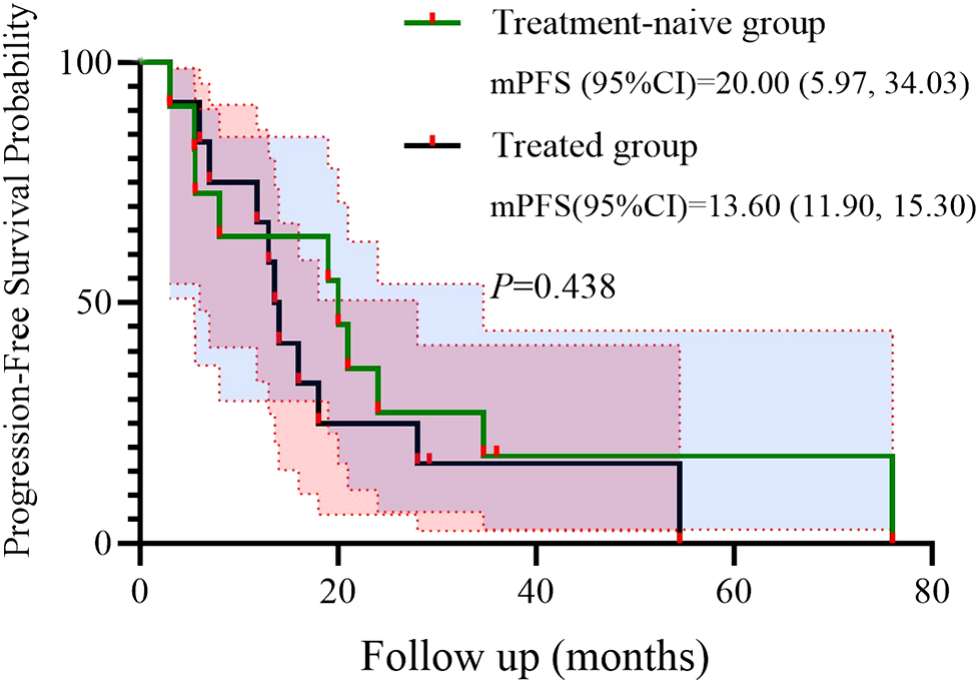
Kaplan–Meier survival curves of PFS stratified by treatment history.

**Figure 5:**
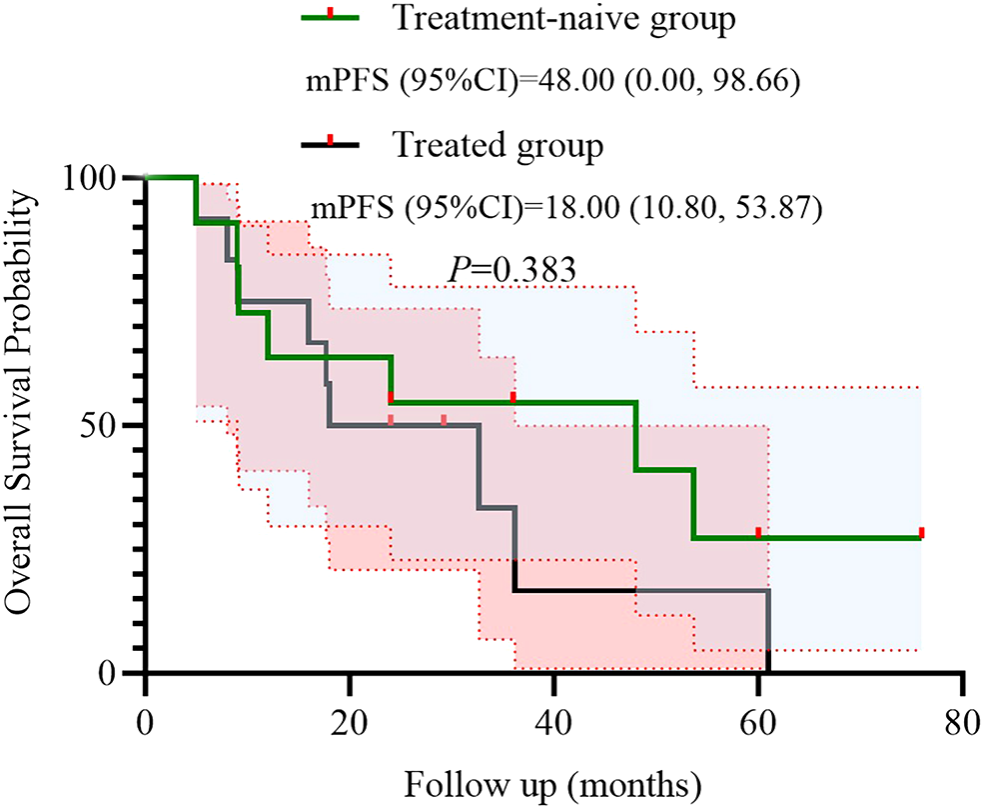
Kaplan–Meier survival curves of OS stratified by treatment history.

We further conducted a subgroup analysis based on prior treatment status. Among the 12 patients who had received systemic therapy before initiating the BRICS regimen, 9 (75.0%) achieved PR, 2 (16.7%) had SD, and 1 (8.3%) showed PD. In the treatment-naïve group (n = 11), 8 (72.7%) achieved PR and 3 (27.3%) had SD, with no cases of PD observed. Kaplan–Meier survival analysis demonstrated that the mPFS was 20.0 months (95% CI: 5.97–34.03) in the treatment-naive group and 13.6 months (95% CI: 11.90–15.30) in the previously treated group (*P* = 0.438) (**Figure 4**). Similarly, the mOS was 48.0 months (95% CI: 0.00–98.66) for treatment-naïve patients and 18.0 months (95% CI: 10.80–53.87) for previously treated patients (*P* = 0.383) (**Figure 5**).

### 3.4 Prognostic factors for PFS and OS

**Figure 6:**
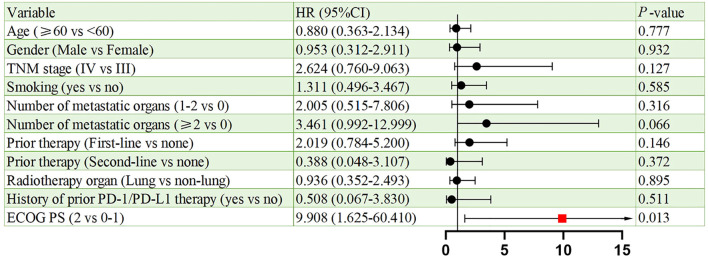
Univariate analysis of prognostic factors for PFS.

**Figure 7:**
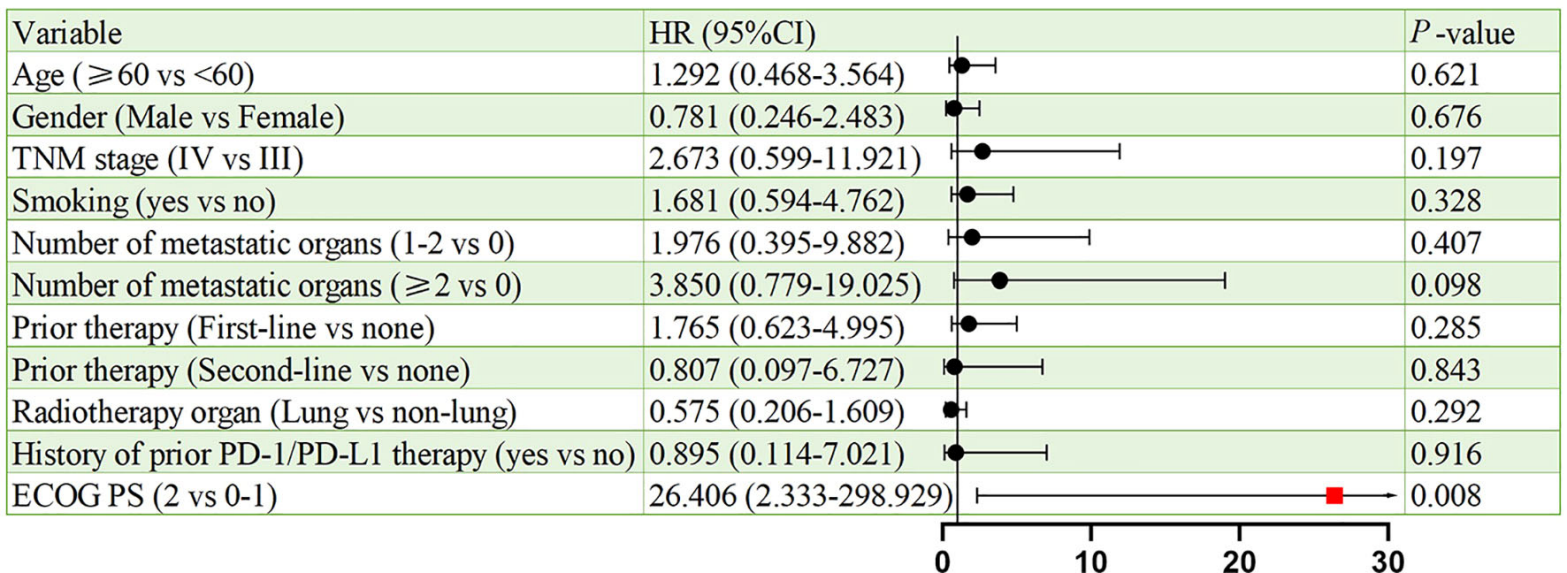
Univariate analysis of prognostic factors for OS.

**Figures 6**, **7** present the analysis results of the prognostic factors for PFS and OS, respectively. The univariate analysis revealed that PS serves as a prognostic factor for both PFS (HR = 9.908, 95% CI: 1.625-60.410, p = 0.013), and OS (HR = 26.406, 95% CI: 2.333-298.929, p = 0.008).

### 3.5 Safety

Adverse events that occurred during the treatment period were presented in the **Table 3**. Adverse events were generally mild and manageable. The most commonly reported treatment-related adverse event was fatigue, occurring in 3 patients (13.2%). Other observed events included rash in 2 patients (8.7%), thrombocytopenia in 2 patients (8.7%), and nausea, diarrhea, anemia, elevated troponin, and elevated creatinine, each in 1 patient (4.3%). No grade 3 or higher adverse events were observed.

**Table 3:** Adverse events.

Adverse events	Grade 1–2, n (%)	Grade 3–4, n (%)
Fatigue	3 (13.2)	0
Nausea	1 (4.3)	0
Diarrhea	1 (4.3)	0
Anemia	1 (4.3)	0
Thrombocytopenia	2 (8.7)	0
Elevated troponin	1 (4.3)	0
Elevated creatinine	1 (4.3)	0
Rash	2 (8.7)	0

## 4 Discussion

The present study was conducted to evaluate the efficacy and safety of the novel “BRICS” sequential therapeutic regimen in patients with EGFR/ALK wild-type, PD-L1-negative advanced NSCLC. Our findings demonstrated potentially remarkable clinical outcomes, including a median PFS of 16 months (95% CI: 9.11–22.89) and a median OS of 32.7 months (95% CI: 11.53–53.87), alongside an ORR of 95.7% and manageable toxicity profiles. In subgroup analysis based on treatment history, patients who received BRICS as first-line therapy demonstrated numerically longer PFS and OS (20.0 vs. 13.6 months for PFS; 48.0 vs. 18.0 months for OS), although the differences were not statistically significant. These results suggest that the BRICS strategy, comprising stereotactic body radiation therapy (SBRT), low-dose chemotherapy, PD-1/PD-L1 inhibitors, and high-dose probiotics, may represent a breakthrough in overcoming the therapeutic bottleneck for PD-L1-negative NSCLC.

PD-L1-negative advanced NSCLC remains a therapeutic challenge, as these patients derive minimal benefit from immune checkpoint inhibitors (ICIs) alone or in combination with chemotherapy. The EXPRESS study reported that nearly 50% of NSCLC patients exhibit PD-L1 tumor proportion scores <1%, correlating with poor responses to ICIs (7). Similarly, the KEYNOTE-189 trial demonstrated that even in PD-L1-negative subgroups, the survival advantage of pembrolizumab-chemotherapy combinations diminished over time, particularly in elderly patients (19). Our cohort, characterized by 100% PD-L1 negativity and a median age of 62 years, achieved a median OS exceeding 32 months, which starkly contrasts with historical data. For instance, real-world studies of pembrolizumab-chemotherapy in PD-L1-negative NSCLC reported median OS durations of 12–18 months (20). This discrepancy highlights the potential of BRICS to recalibrate the tumor microenvironment (TME) and overcome intrinsic resistance mechanisms.

In the present study, the integration of SBRT (8 Gy × 3 fractions) into the BRICS regimen likely plays a pivotal role in enhancing ICI efficacy. Preclinical models have shown that hypo-fractionated radiotherapy promotes antigen release, upregulates MHC-I expression, and induces immunogenic cell death, thereby converting “cold” tumors into “hot” TMEs (21). Clinical evidence from the PEMBRO-RT trial further supports this synergy, where SBRT combined with pembrolizumab doubled the ORR in PD-L1-negative NSCLC compared to ICIs alone (9). Our protocol’s unique timing-administering SBRT before chemotherapy and ICIs—may have primed systemic anti-tumor immunity by increasing tumor-infiltrating lymphocytes (TILs) and dendritic cell activation, as observed in the phase II trial (22). The use of low-dose nab-paclitaxel in BRICS diverges from conventional platinum-based regimens but aligns with emerging evidence supporting metronomic chemotherapy’s immunomodulatory effects. Subtoxic doses of taxanes enhance antigen presentation, deplete regulatory T cells (Tregs), and reduce myeloid-derived suppressor cells (MDSCs), thereby amplifying ICI activity. In the present study, the reduced chemotherapy dosage in BRICS likely mitigated hematologic toxicity, as evidenced by the low incidence of grade ≥3 adverse events (AEs) in our cohort (8.7% thrombocytopenia).

The inclusion of high-dose bifidobacterium/lactobacillus (6 g/day) represents a novel strategy to potentiate ICIs. Mounting evidence links gut microbiome diversity to ICI responsiveness, with *Bifidobacterium* species enhancing dendritic cell maturation and CD8+ T-cell infiltration (23). In the phase I trial, probiotic supplementation increased the efficacy of ICIs in metastatic renal carcinoma (24). Our regimen’s prolonged probiotic administration-throughout the treatment course-may have sustained these benefits, potentially explaining the unprecedented OS observed.

The BRICS regimen shares conceptual parallels with the “PRaG” protocol, which combines SBRT, granulocyte-macrophage colony-stimulating factor (GM-CSF), and ICIs (25–27). However, key distinctions exist: (1) BRICS employs metronomic chemotherapy rather than full-dose cisplatin/pemetrexed, reducing myelosuppression risks; (2) probiotics are administered continuously rather than intermittently; and (3) SBRT targets primary lesions regardless of metastatic burden, whereas PRaG protocols often focus on oligometastatic disease. These modifications may account for BRICS superior tolerability-only 13.2% of patients experienced fatigue, compared to 40%–60% in PRaG regimens. Notably, our results challenge the dogma that PD-L1-negative NSCLC is inherently resistant to ICIs. By leveraging radiotherapy-induced antigen release and microbiome-driven T-cell activation, BRICS may bypass PD-L1 dependency. This aligns with findings from the PRaG trials, where ICIs-CSF-radiotherapy achieved durable responses in PD-L1-negative patients. However, the median PFS and OS in the BRICS exceeds longer than those in PRaG, suggesting additive benefits from probiotics and dose-optimized chemotherapy.

Univariate analysis identified ECOG PS as a negative prognostic factor for both PFS (HR = 9.908; p = 0.013) and OS (HR = 26.406; p = 0.008). This underscores the importance of patient selection, as poor performance status may compromise the ability to tolerate sequential therapies. Interestingly, 91.3% of our cohort had ECOG PS <2, which contrasts with real-world NSCLC populations where 30%–40% exhibit PS ≥2. Future studies should stratify outcomes by PS and explore BRICS feasibility in frailer populations. In addition, a subgroup analysis based on prior treatment status revealed that patients receiving BRICS as first-line therapy showed numerically longer PFS and OS compared to those treated in later lines (20.0 vs. 13.6 months for PFS; 48.0 vs. 18.0 months for OS), although no statistically significant differences were observed. While limited by sample size, this finding may suggest that earlier administration of BRICS could be associated with prolonged disease control, warranting further investigation in prospective cohorts.

The BRICS regimen exhibited a favorable safety profile, with no grade ≥3 AEs reported. This contrasts sharply with traditional chemoimmunotherapy combinations. The low incidence of immune-related AEs (8.7% rash, 4.3% diarrhea) may reflect probiotic-mediated gut barrier stabilization, which has been shown to reduce colitis risk. Additionally, the absence of radiation pneumonitis-a common concern with thoracic SBRT-suggests that targeting primary lesions with strict dose constraints enhanced safety.

This study has several limitations inherent to its retrospective design, including selection bias and heterogeneous radiotherapy targets. The small sample size (n=23) precludes multivariate analysis of confounding variables, such as prior immunotherapy exposure (8.7% of patients). Furthermore, the lack of biomarker data (e.g., TIL density, microbiome sequencing) limits mechanistic insights. Prospective validation in larger cohorts-ideally with correlative translational studies-is warranted to confirm these findings.

In conclusion, the BRICS sequential therapy demonstrates promising efficacy and tolerability in PD-L1-negative advanced NSCLC, a population historically refractory to available treatments. By synergistically modulating the TME through radiotherapy, low-dose chemotherapy, ICIs, and probiotics, this regimen may redefine therapeutic paradigms for immunologically “cold” tumors. Subgroup analysis showed numerically longer PFS and OS in patients receiving BRICS as first-line therapy, suggesting potential benefits with earlier intervention, although further validation is needed. The prospective trial is ongoing. With the process, our findings may provide the potential of multimodal immunomodulation to overcome resistance mechanisms in NSCLC.

## Data Availability

The raw data supporting the conclusions of this article will be made available by the authors, without undue reservation.
